# Clinically Relevant Patterns of Co‐Fluctuating Structure and Function in Multiple Sclerosis

**DOI:** 10.1111/ene.70367

**Published:** 2025-09-29

**Authors:** Jian Zhang, Marco Battaglini, Rosa Cortese, Maria Laura Stromillo, Marzia Mortilla, Ludovico Luchetti, Giordano Gentile, Emilio Portaccio, Maria Pia Amato, Menno M. Schoonheim, Nicola De Stefano

**Affiliations:** ^1^ Department of Medicine, Surgery and Neuroscience University of Siena Siena Italy; ^2^ SIENA Imaging SRL Siena Italy; ^3^ Radiology Anna Meyer Children's University Hospital‐ IRCCS Florence Italy; ^4^ Department of Neurofarba University of Florence Florence Italy; ^5^ IRCCS Fondazione Don Carlo Gnocchi Florence Italy; ^6^ MS Center Amsterdam, Anatomy and Neurosciences, Vrije Universiteit Amsterdam, Amsterdam Neuroscience Amsterdam UMC Location VUmc Amsterdam the Netherlands

**Keywords:** co‐fluctuating patterns, functional hyperconnectivity, multimodal MRI, multiple sclerosis, structural disconnection

## Abstract

**Background:**

While structural and functional connectivity changes in multiple sclerosis (MS) are well documented, their complex interplay remains poorly understood. This study identifies co‐fluctuating patterns of structural and functional changes in MS using an MRI‐based multimodal fusion approach and assesses the added value to clinical outcomes.

**Methods:**

Linked independent component analysis (ICA) was applied to spatial maps of white matter (WM) lesions, fractional anisotropy (FA), gray matter (GM) volume, and functional network connectivity to detect regions with differential co‐fluctuations. Graph theory (GT) was then used to reveal clusters of interconnected brain regions. Linear mixed‐effect models targeted regions with significantly different co‐fluctuating patterns between MS and HC. Multivariate stepwise regressions analyzed the associations between co‐fluctuating patterns and disability and cognitive dysfunction.

**Results:**

The study included 147 patients with MS and 57 HC. Significant co‐fluctuating patterns of decreased FA, increased lesion probability in the thalamic radiation and corpus callosum, GM atrophy in sensorimotor and thalamic areas, and enhanced functional connectivity in the temporal parietal network distinguished MS from HC (*p* < 0.001). GT revealed eight brain sub‐networks of spatially connected clusters. Regional‐ and modality‐specific loadings and GT changes explained physical (adjusted *R* ^2^ = 0.51, *p* < 0.001) and cognitive (adjusted *R* ^2^ = 0.44, *p* < 0.001) disability better than traditional MRI measures (adjusted *R* ^2^ = 0.12–0.33, *p* < 0.001).

**Conclusions:**

Our multimodal MRI approach revealed co‐fluctuating regional patterns of lesions, structural disconnection, and functional hyperconnectivity in MS, offering a more comprehensive explanation of clinical outcomes than traditional MRI metrics.

## Graphical abstract

Multimodal component analysis identified co‐fluctuating regional patterns of lesions, structural disconnection, and hyperconnectivity in MS, providing superior explanations of clinical outcomes compared to traditional MRI metrics.

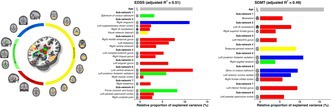

## 1 Introduction

Multiple sclerosis (MS) is a demyelinating and neurodegenerative disease of the central nervous system [1]. Pathological changes in MS include both focal areas of demyelination and widespread neuroaxonal damage. Focal and diffuse brain tissue damage accumulates over time [2] and leads to progressive physical and cognitive disability. Both are believed to arise, at least in part, from a collapsing network due to tissue damage exceeding a certain threshold beyond which crucial network efficiency is lost [3]. Over the years, numerous neuroimaging studies have investigated these pathological abnormalities using conventional and non‐conventional MRI techniques to characterize which measures best explain complex symptoms [4]. However, when used individually, each MRI modality has substantially failed to capture the full spectrum of changes in the MS brain [4]. Efforts to integrate measures of focal and diffuse damage have yielded only marginal improvements [5].

An important reason for these failings could lie in the common exclusion of functional alterations, which are thought to arise as a response to structural disconnection [4]. Initially, it was hypothesized that the frequently observed hyperconnectivity might serve as a compensatory mechanism [6]. However, subsequent findings indicated that these changes often signify a deteriorating network, possibly due to a loss of inhibitory control [6]. Recently, researchers have focused on innovative methods to integrate function and structure to grasp both the damage itself and the way the brain responds to this, introducing multilayered concepts of structure–function coupling [7] that could offer a more realistic representation of the brain [7]. Preliminary studies have shown a decoupling between structure and function as patients progress in very early MS [8]. However, a stronger coupling has been observed in later stages, particularly when cognitive impairment is prevalent [9]. However, this methodology requires alignment of possible connections within the same network, which could lead to the exclusion of relevant information linking different types of modalities. More recently, linked independent component analysis (ICA) has been introduced to concurrently assess structural‐functional damage in a unified analysis without the need for a shared spatial topology [10]. This approach thus possibly provides a more comprehensive understanding of spatially both overlapping and non‐overlapping neurobiological changes [10]. In MS, this approach remains rare, but recent studies have used linked ICA to identify covaried white matter (WM) microstructural damage and gray matter (GM) atrophy in Huntington's disease [11], linked WM microstructural damage and functional network reorganization after brain concussion [12], and connected WM microstructural damage, GM atrophy, and functional network reorganization in attention‐deficit/hyperactivity disorder [13].

In this study, we conducted an integrated multimodal linked ICA analysis to examine patterns of structural damage (including WM lesions, microstructural damage to tracts, and GM atrophy) and altered functional connectivity (FC). In addition, graph theory (GT) was used to further investigate network hierarchy within the linked multimodal components.

Given the growing interest in multimodal data fusion, it is important to consider how our approach compares with existing techniques. While joint ICA [14], multilayer networks [7], and Graph Neural Networks (GNNs) [14] offer powerful data fusion capabilities, each is not without limitations—joint ICA assumes a shared mixing matrix, potentially limiting modality‐specific sensitivity, whereas multilayer networks and GNNs often rely on predefined parcellations, which may introduce bias. In contrast, our fully data‐driven framework combines hierarchical ICA and GT to fuse multimodal features—including hypointense lesions, an established marker of irreversible MS damage—within a unified statistical model without such constraints.

Building on this framework, we aimed to identify the clinical relevance of such co‐fluctuating patterns of structural and functional changes in MS compared to more conventional global MRI measures.

## 2 Materials and Methods

This retrospective study was approved by the Ethics Committee of Azienda Ospedaliera Universitaria Senese. Informed written consent was obtained from all participants.

### 2.1 Study Participants

Between 2012 and 2019, we recruited MS patients from the MS Centres of the University Hospital of Siena and Florence, Tuscany, Italy. Inclusion criteria were: (1) diagnosis of MS according to the 2017 McDonald criteria [1]; (2) freedom from relapses and corticosteroid treatment for at least 1 month before study entry; and (3) no major contraindications to MRI. Clinical disability was assessed with the expanded disability status score (EDSS) [15]. Information processing speed was assessed using the symbol digit modality test (SDMT) [16]. Sex‐matched healthy controls (HC) were also recruited, with a normal neurological examination and no history of neurological or psychiatric disorder.

### 2.2 MRI Data Acquisition

All participants were scanned on a 3 T MRI scanner (Philips Medical Systems, Best, The Netherlands) located at Meyer University Hospital, Florence. The brain MRI acquisition protocol is described in the Supporting Information.

### 2.3 MRI Analysis

#### 2.3.1 Quality Control

All images were visually inspected for artifacts or incidental findings. Those of inadequate quality were excluded from further analysis.

#### 2.3.2 Multimodal Imaging Fusion

First, we extracted representative MRI features: WM lesions and fractional anisotropy (FA), GM atrophy with VBM, and FC of resting state networks (RSNs) based on fMRI (details in Supporting Information). Next, the normalized spatial maps of lesions, FA, GM‐VBM, and FC were merged to identify co‐fluctuating patterns by linked ICA, a data‐driven approach that can leverage cross‐modality information and automatically discover meaningful patterns in voxel‐level multimodal data [10]. To prevent overfitting, a model order less than 25% of the sample size is generally recommended [10]. In this case, after 1000 iterations, 20 independent components (ICs) were identified across modalities and across the study populations (MS and HC) [10]. Participant‐specific IC loading coefficients were calculated (See Supporting Information for details as well).

#### 2.3.3 Multimodal Nodes Extraction

The data were then converted to graphs of nodes (brain regions) and edges (cross‐region and cross‐modal relations), enabling the computation of graph theoretical parameters to explore network hierarchy within significant linked ICA components (See Supporting Information for details).

### 2.4 Statistical Analysis

Between‐group comparisons of demographic and clinical variables were performed using the *χ* ^2^, 2‐sample *t*‐test, or Mann–Whitney *U* test, as appropriate.

#### 2.4.1 Aim 1: Identifying Linked Multimodal Networks That Differ Between MS and HC

Linear mixed‐effect models were used to compare the participant loading coefficients of the ICs between MS and HC, with age and sex as fixed‐effect nuisance variables (*p* < 0.05, False Discovery Rate [FDR] corrected). This analysis was performed using the R software v3.6.3 (https://www.r‐project.org/).

#### 2.4.2 Aim 2: Identification of Linked Structural and Functional Sub‐Networks

For each region and modality, two indices are provided: the participation coefficient (PC) and the within‐community (WC) strength coefficient, respectively reflecting how evenly distributed a node's edges are across communities and how strong is its correlation is with the other regions and modalities within the same community (See Supporting Information for mathematical definition). We named “connector hub” the region of each identified subnetwork with bigger PC and “local hub” the regions with higher WC.

Then using these node weights, we created a partial correlation matrix using Gaussian graphical models (GGM), with the values of partial correlation used to create the edges of our GT (See Supporting Information for full procedure).

#### 2.4.3 Aim 3: Assessing Associations Between Linked Structural and Functional Sub‐Networks and Clinical Scores

Multivariate stepwise linear regression selection was used to identify potential predictors of clinical outcomes (EDSS and SDMT) from regional‐ and modality‐specific averaged loading coefficients, together with age and sex.

The same approach was also used to identify the best traditional MRI predictors. Four stepwise regression models were run using age and sex with (1) global lesion volume, (2) regional brain volumes, (3) tract‐specific mean FA values, and (4) functional network connectivity as independent predictors, respectively (See Table S1 for a complete list). Then, the variables that survived the initial analyses were combined in a final stepwise regression to evaluate the collective efficacy of independently derived unimodal MRI measures in explaining EDSS and SDMT.

Finally, for a quantitative comparison of the best selected models—one based on regional and modality‐specific average loading coefficients and the other using a combination of global lesion volume, regional brain volumes, tract‐specific mean FA, and functional network connectivity—we computed a performance score for each model (See Supporting Information for details).

## 3 Results

### 3.1 Participant Characteristics

Data from 147 patients with MS (mean age, 41.0 years ±10.3 [standard deviation], 106 women) and 57 HC (mean age, 35.6 years ±9.5 [standard deviation], 36 women) were collected, where the MS patients included 137 with relapsing–remitting MS (RRMS), 3 with secondary‐progressive MS (SPMS), and 7 with primary‐progressive MS (PPMS). Demographic and clinical data are summarized in Table 1.

**TABLE 1:** Clinical, demographic and conventional MRI characteristics of the study groups.

	MS patients (*n* = 147)	HC (*n* = 57)	*p*
Age (mean ± SD, y)	41.0 ± 10.3	35.6 ± 9.5	< 0.001^a^
Sex (men/women no.)	41/106	21/36	0.212^b^
Disease duration (mean ± SD, y)	10.2 ± 7.5	—	—
EDSS (median (range))	1.5 (0–6.5)	—	—
SDMT (*n* = 100, mean ± SD)	49.1 ± 11.8	—	—
Lesion			
LV (median (Q1–Q3), cm^3^)	3.46 (1.17–7.72)	—	—
Brain volumes			
NBV (mean ± SD, cm^3^)	1330.1 ± 49.4	1365.8 ± 41.1	< 0.001^a^
NGMV (mean ± SD, cm^3^)	743.2 ± 43.4	770.2 ± 35.7	< 0.001^a^
NPGMV (mean ± SD, cm^3^)	552.2 ± 36.4	568.6 ± 30.3	0.001^a^
NSGMV (mean ± SD, cm^3^)	53.5 ± 6.1	59.6 ± 3.7	< 0.001^a^
Atlas‐based mean FA values			
Middle cerebellar peduncle	0.49 ± 0.03	0.52 ± 0.02	< 0.001^a^
Pontine crossing tract	0.43 ± 0.03	0.46 ± 0.03	< 0.001^a^
Genu of corpus callosum	0.50 ± 0.04	0.53 ± 0.02	< 0.001^a^
Body of corpus callosum	0.54 ± 0.05	0.59 ± 0.02	< 0.001^a^
Splenium of corpus callosum	0.65 ± 0.05	0.70 ± 0.02	< 0.001^a^
Fornix (column and body of fornix)	0.31 ± 0.06	0.38 ± 0.05	< 0.001^a^
Corticospinal tract R	0.56 ± 0.05	0.58 ± 0.03	< 0.001^a^
Corticospinal tract L	0.56 ± 0.04	0.58 ± 0.04	0.001^a^
Medial lemniscus R	0.55 ± 0.04	0.57 ± 0.03	< 0.001^a^
Medial lemniscus L	0.55 ± 0.04	0.57 ± 0.03	< 0.001^a^
Inferior cerebellar peduncle R	0.47 ± 0.04	0.50 ± 0.03	< 0.001^a^
Inferior cerebellar peduncle L	0.47 ± 0.04	0.50 ± 0.03	< 0.001^a^
Superior cerebellar peduncle R	0.51 ± 0.04	0.53 ± 0.03	< 0.001^a^
Superior cerebellar peduncle L	0.49 ± 0.03	0.51 ± 0.02	< 0.001^a^
Cerebral peduncle R	0.60 ± 0.03	0.64 ± 0.02	< 0.001^a^
Cerebral peduncle L	0.60 ± 0.03	0.64 ± 0.02	< 0.001^a^
Anterior limb of internal capsule R	0.50 ± 0.03	0.52 ± 0.02	< 0.001^a^
Anterior limb of internal capsule L	0.49 ± 0.03	0.51 ± 0.02	< 0.001^a^
Posterior limb of internal capsule R	0.58 ± 0.03	0.61 ± 0.02	< 0.001^a^
Posterior limb of internal capsule L	0.59 ± 0.03	0.61 ± 0.02	< 0.001^a^
Retrolenticular part of internal capsule R	0.51 ± 0.04	0.55 ± 0.02	< 0.001^a^
Retrolenticular part of internal capsule L	0.52 ± 0.04	0.56 ± 0.02	< 0.001^a^
Anterior corona radiata R	0.38 ± 0.04	0.42 ± 0.02	< 0.001^a^
Anterior corona radiata L	0.38 ± 0.04	0.41 ± 0.02	< 0.001^a^
Superior corona radiata R	0.41 ± 0.03	0.44 ± 0.02	< 0.001^a^
Superior corona radiata L	0.41 ± 0.03	0.44 ± 0.02	< 0.001^a^
Posterior corona radiata R	0.40 ± 0.04	0.45 ± 0.02	< 0.001^a^
Posterior corona radiata L	0.39 ± 0.04	0.44 ± 0.02	< 0.001^a^
Posterior thalamic radiation R	0.50 ± 0.06	0.56 ± 0.03	< 0.001^a^
Posterior thalamic radiation L	0.48 ± 0.06	0.54 ± 0.02	< 0.001^a^
Sagittal stratum R	0.46 ± 0.05	0.51 ± 0.02	< 0.001^a^
Sagittal stratum L	0.43 ± 0.04	0.48 ± 0.02	< 0.001^a^
External capsule R	0.36 ± 0.03	0.38 ± 0.01	< 0.001^a^
External capsule L	0.37 ± 0.03	0.39 ± 0.02	< 0.001^a^
Cingulum (cingulate gyrus) R	0.42 ± 0.04	0.46 ± 0.02	< 0.001^a^
Cingulum (cingulate gyrus) L	0.45 ± 0.04	0.50 ± 0.02	< 0.001^a^
Cingulum (hippocampus) R	0.34 ± 0.03	0.37 ± 0.03	< 0.001^a^
Cingulum (hippocampus) L	0.33 ± 0.04	0.36 ± 0.03	< 0.001^a^
Fornix (crus)/Stria terminalis R	0.40 ± 0.06	0.46 ± 0.03	< 0.001^a^
Fornix (crus)/Stria terminalis L	0.43 ± 0.05	0.48 ± 0.02	< 0.001^a^
Superior longitudinal fasciculus R	0.43 ± 0.03	0.46 ± 0.02	< 0.001^a^
Superior longitudinal fasciculus L	0.43 ± 0.03	0.46 ± 0.02	< 0.001^a^
Superior fronto‐occipital fasciculus R	0.42 ± 0.04	0.46 ± 0.03	< 0.001^a^
Superior fronto‐occipital fasciculus L	0.40 ± 0.05	0.45 ± 0.03	< 0.001^a^
Uncinate fasciculus R	0.41 ± 0.04	0.44 ± 0.03	< 0.001^a^
Uncinate fasciculus L	0.40 ± 0.04	0.41 ± 0.03	< 0.001^a^
Tapetum R	0.34 ± 0.05	0.39 ± 0.03	< 0.001^a^
Tapetum L	0.28 ± 0.04	0.32 ± 0.03	< 0.001^a^
Functional network connectivity			
Visual network (medial)	0.08 ± 1.03	−0.19 ± 0.90	0.084^a^
Default mode network	−0.03 ± 1.01	0.08 ± 0.98	0.490^a^
Visual network (lateral)	0.09 ± 0.99	−0.22 ± 1.00	0.051^a^
Executive control network	0.02 ± 1.06	−0.05 ± 0.84	0.652^a^
Visual network (occipital pole)	0.05 ± 1.01	−0.12 ± 0.99	0.288^a^
Temporal parietal network	0.07 ± 1.08	−0.19 ± 0.73	0.089^a^
Sensorimotor network	0.05 ± 1.01	−0.13 ± 0.98	0.249^a^
Left frontoparietal network	0.03 ± 1.00	−0.09 ± 1.02	0.431^a^
Right frontoparietal network	0.03 ± 0.99	−0.09 ± 1.03	0.443^a^
Cerebellar network	0.10 ± 1.08	−0.25 ± 0.72	0.025^a^
Subcortical network	0.04 ± 1.08	−0.09 ± 0.76	0.424^a^

Abbreviations: EDSS, expanded disability status scale; FA, fractional anisotropy; HC, healthy control; L, left; LV, lesion volume; MS, multiple sclerosis; NBV, normalized brain volume; NGMV, normalized gray matter volume; NPGMV, normalized peripheral gray matter volume; NSGMV, normalized subcortical gray matter volume; Q1, first quartile; Q3, third quartile; R, right; SD, standard deviation; SDMT, symbol digit modalities test.

^a^ Two‐sample *t*‐test.

^b^ Chi‐squared test.

The cohort was moderately disabled with a median EDSS of 1.5 and an SDMT score of 49.1. The cohort showed significantly reduced FA, low GM volumes, and increased FC compared to HC (Table 1).

### 3.2 Differences in Linked Multimodal Brain Networks Between MS and HC

**FIGURE 1:**
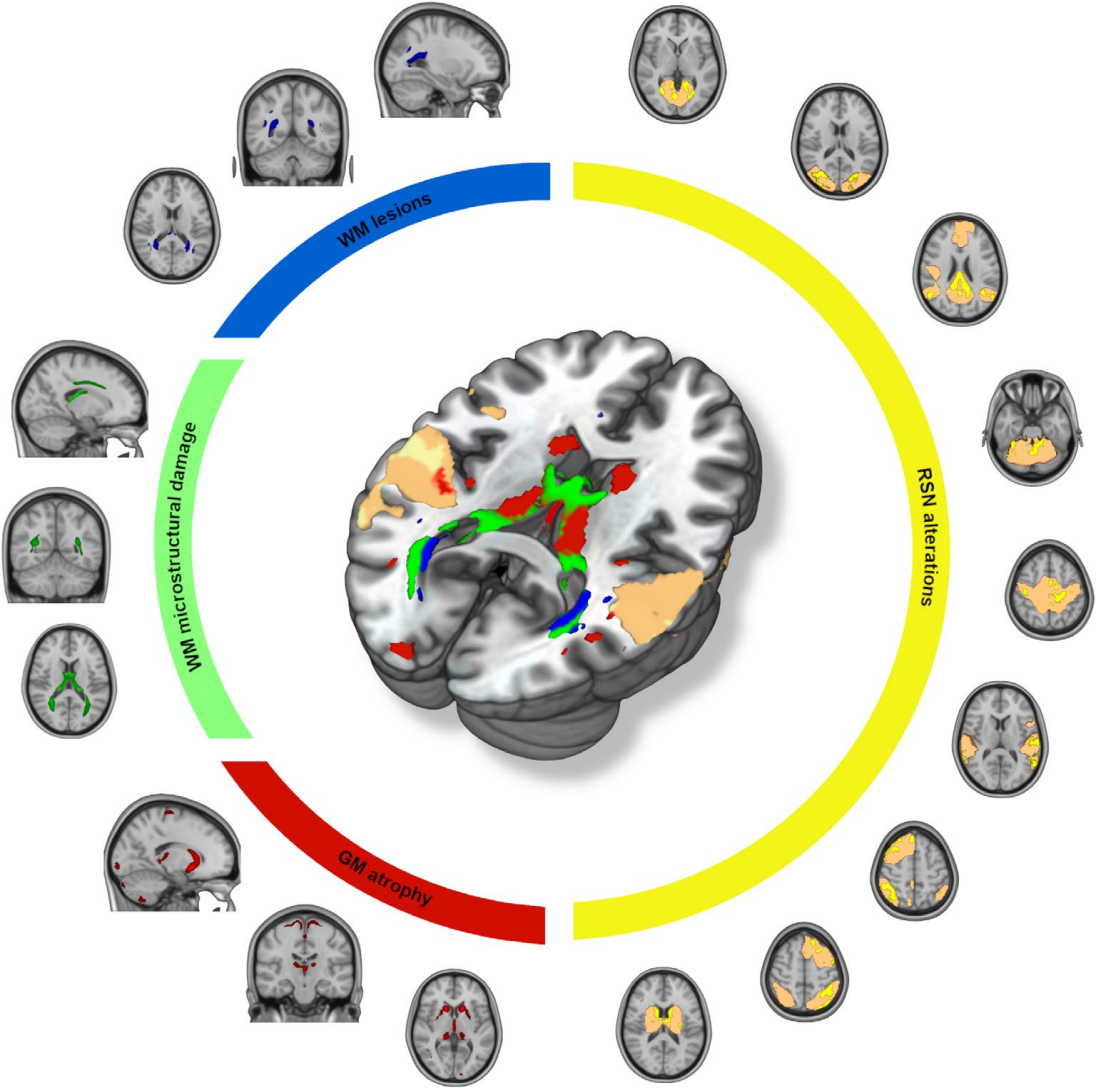
Spatial representation of each modality's contribution to the model. In MS, a pathological change in brain network was found when compared to HC. In particular, MS exhibited involvement WM lesions (blue) and microstructural damage (green) located mainly in the proximity of the posterior horns of the lateral ventricles, atrophy (red) in the cortical GM (precentral, postcentral and occipital gyrus), subcortical GM and cerebellar cortex, and widespread RSN alterations (copper: Networks; yellow: Significant alterations compared to HC). GM, gray matter; HC, healthy controls; MS, multiple sclerosis; RSN, resting state network; WM, white matter.

Among the 20 ICs from linked ICA, MS patients showed one distinct multimodal pattern compared to HC (*p* < 0.01, FDR corrected). This pattern included: (i) WM lesions and reduced FA located mainly in the central and posterior WM regions; (ii) atrophy in the cortical GM (precentral, postcentral, and occipital gyrus), subcortical GM (caudate, putamen, and thalamus), and cerebellar cortex; (iii) widespread increased within‐network FC (medial and lateral visual network, default mode network, cerebellar network, sensorimotor network, temporal parietal network, right frontoparietal network, left frontoparietal network, subcortical network) (Figure 1).

### 3.3 Identification of Linked Structural and Functional Sub‐Networks

**FIGURE 2:**
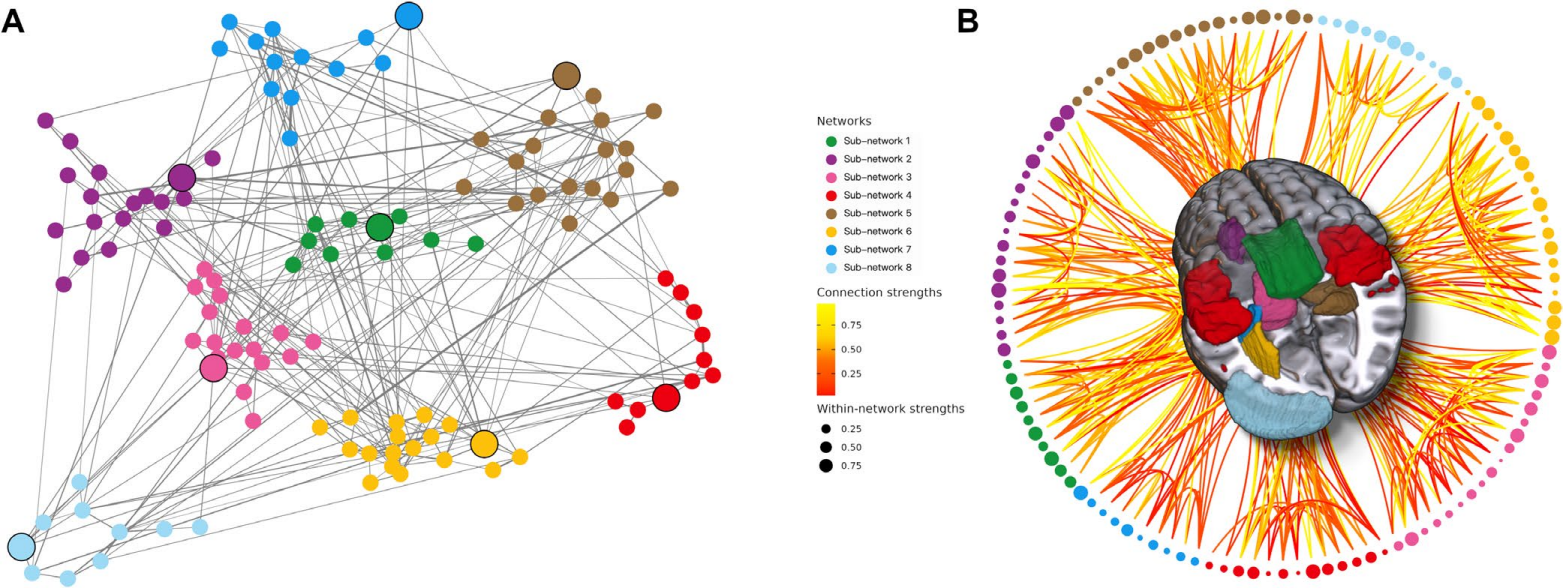
Pathological change of brain network architecture in MS brain. The 8 structural and functional brain subnetworks which mostly contributed to the model are shown in the figure. Each subnetwork is represented with a different color in a schematic representation (A) and projected onto a 3D brain (B). The connector hubs (nodes) and for each network is presented with bigger size and black border. For each network, the bigger size of a node means higher within‐community strength, indicating higher connections within their own network. The 8 sub‐networks were colored and designated as follows for both A and B: (1) green pantone: Central WM lesion mediated sub‐network (sub‐network 1); (2) vivid purple: Left anterior ventricular WM microstructural damage mediated sub‐network (sub‐network 2); (3) bright sun yellow: Left posterior ventricular WM microstructural damage mediated sub‐network (sub‐network 6); (4) vivid sky blue: Left lateral ventricular WM microstructural damage mediated sub‐network (sub‐network 7); (5) light fuchsia pink: Left subcortical GM atrophy mediated sub‐network (sub‐network 3); (6) coyote brown: Right subcortical GM atrophy mediated sub‐network (sub‐network 5); (7) bright lemon yellow: Left cerebellar atrophy mediated sub‐network (sub‐network 8); (8) bright red: Temporal RSN activity alteration mediated sub‐network (sub‐network 4). For (B), connection strengths are represented with a red‐yellow color gradient, where colors closer to red indicate lower connection strengths and colors closer to yellow indicate higher connection strengths; within‐network strengths are represented by the diameter of the black ball, where a larger diameter indicates stronger within‐network strengths.

From the spatial maps of the single Linked ICA component, the Louvain method detected eight sub‐networks from the identified nodes (Figure 2) with *Q* = 0.37. For each sub‐network, GT analysis was employed to derive indices, through which both the connector and the local hub [17] were identified based on PC and WC for each region of a community (Table 2, Figure 2). The anatomical location of the connector hub within each sub‐network was used to name each sub‐network, namely (1) central WM lesions, (2–4) left anterior and posterior and left lateral periventricular WM microstructural damage, (5–7) left and right subcortical and cerebellar atrophy, and (8) increased temporal FC. See Table S2 for the complete list of the sub‐networks.

**TABLE 2:** Detected pathological changes of brain network architecture in multiple sclerosis.

Sub‐network	Connector hub	Local hub
Central WM lesion mediated network	Body of corpus callosum (lesion, ↑)	Right cerebral peduncle (lesion, ↑) Brain‐Stem (GM, ↓) Visual network (medial) (RSN, ↑)
Left anterior ventricular WM microstructural damage mediated network	Left anterior corona radiata (FA, ↓)	Right cingulum (lesion, ↑) Left superior corona radiata (FA, ↓) Right juxtapositional lobule (GM, ↓) Left IX cerebellum (GM, ↓) Left VIIIb cerebellum (GM, ↓)
Left posterior ventricular WM microstructural damage mediated network	Left posterior thalamic radiation (FA, ↓)	Left ventral striatum (GM, ↓)
Left lateral ventricular WM microstructural damage mediated network	Left retrolenticular part of internal capsule (FA, ↓)	Left cingulum (lesion, ↑) Right thalamus (GM, ↓)
Left subcortical GM atrophy mediated network	Left thalamus (GM, ↓)	Left planum temporale (GM, ↓) Left putamen (GM, ↓)
Right subcortical GM atrophy mediated network	Right hippocampus (GM, ↓)	Right posterior thalamic radiation (FA, ↓)
Left cerebellar atrophy mediated network	Left crus II cerebellum (GM, ↓)	Fornix (FA, ↓) Subcortical network (RSN, ↑)
Temporal RSN activity alteration mediated network	Temporal parietal network (RSN, ↑)	Left precentral gyrus (GM, ↓) Right lingual gyrus (GM, ↓) Right frontoparietal network (RSN, ↑)

*Note:* ↑: compared to healthy controls, multiple sclerosis patients presented increased lesions and resting state network activity in the hubs; ↓: compared to healthy controls, multiple sclerosis patients presented decreased gray matter volume and white matter fractional anisotropy in the hubs.

Abbreviations: GM, gray matter; RSN, resting state network; WM, white matter.

### 3.4 Associations Between Identified Linked Structural and Functional Sub‐Networks and Clinical Disability in MS

**FIGURE 3:**
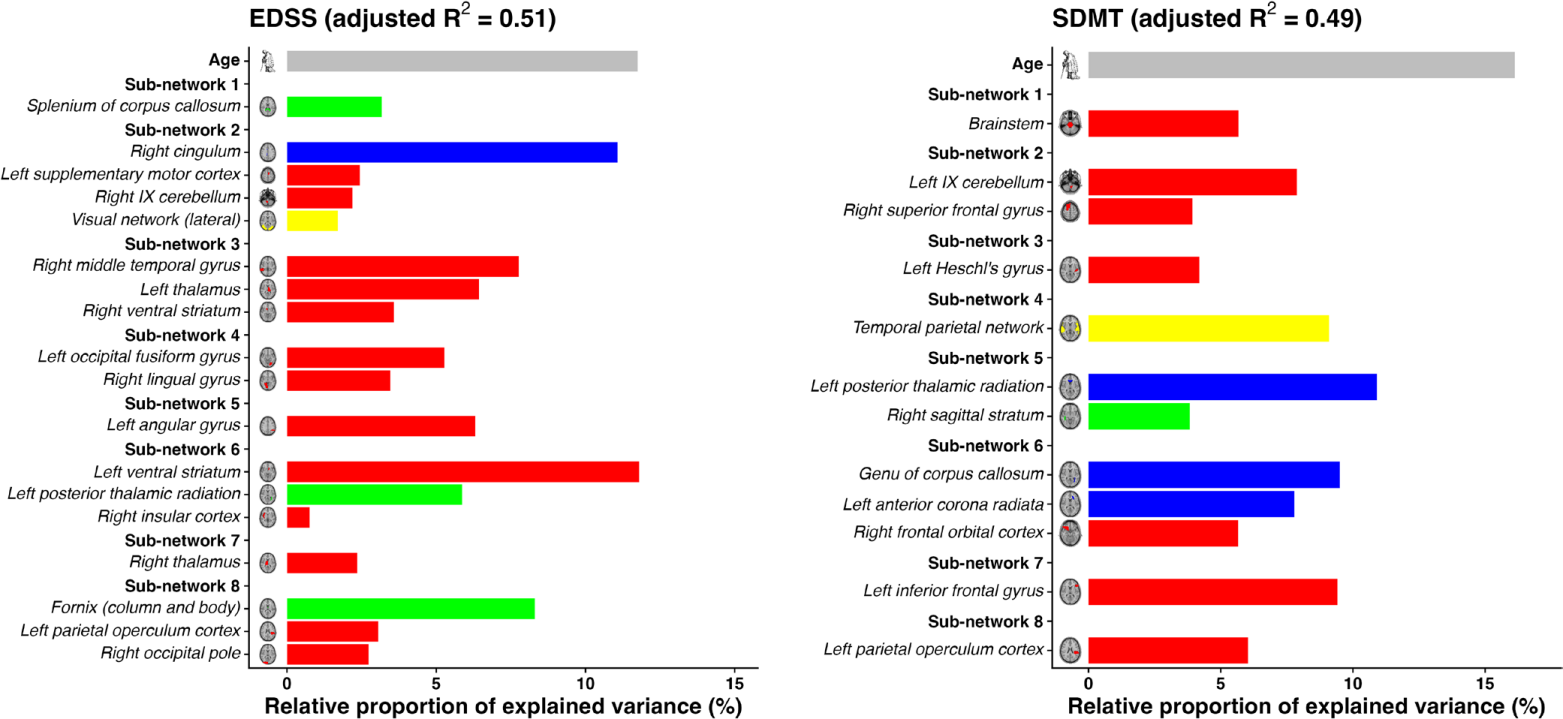
Relationships between the identified multimodal pattern and clinical disability in MS patients. The bar plots represent the association of each component of the model with Expanded Disability Status Scale (EDSS) (left) and Symbol Digit Modalities Test (SDMT) (right). The bar plots for MRI measures are ordered to reflect their relative proportion of explained variance (%) for EDSS (left) and SDMT (right). The color assigned to the y‐axis for a predictor denotes the specific sub‐network associated with each MRI measure. The color of the bar plot indicates the modality: Blue: white matter lesion; Red: gray matter volume; green: White matter fractional anisotropy; Yellow: resting state network. A bar plot for Age was also represented.

Table 3, Table 4 and Figure 3 summarize the results of multivariate stepwise regression for EDSS and SDMT using predictors from the regional‐ and modality‐specific averaged loading coefficients obtained by ICA analysis. In particular, we found that:

**TABLE 3:** Predictors of identified multimodal pattern in explaining expanded disability status scale (EDSS) and symbol digit modalities test (SDMT).

Predictor	β coefficient	*p*	Relative proportion of explained variance (%)
EDSS (*R* ^2^ = 0.58, adjusted *R* ^2^ = 0.51, *n* = 147)		< 0.001	
Left ventral striatum GM	−14.68	< 0.001	11.8
Age	0.03	< 0.001	11.7
Right cingulum lesion	13.44	< 0.001	11.1
Fornix (column and body of fornix) FA	−21.22	0.022	8.3
Right middle temporal gyrus GM	8.38	< 0.001	7.8
Left thalamus GM	−20.39	0.005	6.4
Left angular gyrus GM	−4.18	0.001	6.3
Left posterior thalamic radiation FA	−24.07	0.004	5.9
Left occipital fusiform gyrus GM	−4.29	0.062	5.3
Right ventral striatum GM	15.28	< 0.001	3.6
Right lingual gyrus GM	−1.88	0.001	3.5
Splenium of corpus callosum FA	−16.21	0.02	3.2
Left parietal operculum cortex GM	5.02	0.014	3.1
Right occipital pole GM	4.29	0.004	2.7
Left supplementary motor cortex GM	−1.95	0.017	2.4
Right thalamus GM	12.00	0.037	2.4
Right IX cerebellum GM	4.82	0.003	2.2
Visual network (lateral)	1.49	0.012	1.7
Right insular cortex GM	3.18	0.092	0.8
SDMT (*R* ^2^ = 0.55, adjusted *R* ^2^ = 0.49, *n* = 100)		< 0.001	
Age	−0.35	< 0.001	16.1
Left posterior thalamic radiation lesion	−169.48	0.001	10.9
Genu of corpus callosum lesion	−30.22	0.241	9.5
Left inferior frontal gyrus GM	291.53	< 0.001	9.4
Temporal parietal network	16.45	0.005	9.1
Left IX cerebellum GM	44.54	0.024	7.9
Left anterior corona radiata lesion	−100.68	0.089	7.8
Left parietal operculum cortex GM	−47.77	0.008	6.0
Brain‐Stem GM	−72.06	< 0.001	5.7
Right frontal orbital cortex GM	19.90	0.009	5.7
Left Heschl's gyrus GM	2.49	0.07	4.2
Right superior frontal gyrus GM	−6.45	0.037	3.9
Right sagittal stratum FA	−160.70	0.003	3.8

*Note:* Predictors were ordered based on their explained *R* ^2^ for EDSS and SDMT, respectively. The analysis was performed only for the 100 MS patients with assessed SDMT. Network label: (1) central WM lesion mediated network; (2) left anterior ventricular WM microstructural damage mediated network; (3) left posterior ventricular WM microstructural damage mediated network; (4) left lateral ventricular WM microstructural damage mediated network; (5) left subcortical GM atrophy mediated network; (6) right subcortical GM atrophy mediated network; (7) left cerebellar atrophy mediated network; (8) temporal RSN activity alteration mediated network.

Abbreviations: FA, fractional anisotropy; GM, gray matter.

**TABLE 4:** Predictors of traditional MRI measures in explaining expanded disability status scale (EDSS) and symbol digit modalities test (SDMT).

Predictor	β coefficient	*p*
EDSS (*n* = 147)		
LV + Atrophy + FA + RSN (*R* ^2^ = 0.37, adjusted *R* ^2^ = 0.33)		< 0.001
Normalized brain volume	−0.005	0.087
Right inferior cerebellar peduncle mean FA	−8.924	0.025
Age	0.03	0.008
Right superior cerebellar peduncle mean FA	−8.71	0.051
Right superior longitudinal fasciculus mean FA	26.876	< 0.001
Right external capsule mean FA	−20.739	0.002
Right uncinate fasciculus mean FA	10.524	0.014
Splenium of corpus callosum mean FA	−8.978	0.052
WM LV (*R* ^2^ = 0.13, adjusted *R* ^2^ = 0.12)		< 0.001
Age	0.042	< 0.001
T2 LV (log)	0.151	0.061
Atrophy (*R* ^2^ = 0.18, adjusted *R* ^2^ = 0.17)		< 0.001
Normalized brain volume	−0.009	< 0.001
Age	0.020	0.089
Atlas based FA (*R* ^2^ = 0.35, adjusted *R* ^2^ = 0.32)		< 0.001
Right inferior cerebellar peduncle mean FA	−10.161	0.01
Age	0.039	< 0.001
Right superior cerebellar peduncle mean FA	−8.655	0.054
Right superior longitudinal fasciculus mean FA	27.069	< 0.001
Splenium of corpus callosum mean FA	−10.637	0.019
Right external capsule mean FA	−20.247	0.003
Right uncinate fasciculus mean FA	10.237	0.018
RSN (no significant predictor)		—
SDMT (*n* = 100)		
LV + Atrophy + FA + RSN (*R* ^2^ = 0.29, adjusted *R* ^2^ = 0.25)		< 0.001
Left superior corona radiata mean FA	81.429	0.123
Age	−0.355	0.002
Right external capsule mean FA	−180.595	0.001
Right cingulum mean FA	84.983	0.058
Left superior cerebellar peduncle mean FA	95.376	0.068
WM LV (*R* ^2^ = 0.18, adjusted *R* ^2^ = 0.16)		< 0.001
Age	−0.357	0.001
T2 LV (log)	−2.376	0.010
Atrophy (*R* ^2^ = 0.15, adjusted *R* ^2^ = 0.13)		< 0.001
Normalized GM volume	0.096	< 0.001
Atlas based FA (*R* ^2^ = 0.29, adjusted *R* ^2^ = 0.25)		< 0.001
Left superior corona radiata mean FA	81.429	0.123
Age	−0.355	0.002
Right external capsule mean FA	−180.595	0.001
Right cingulum mean FA	84.983	0.058
Left superior cerebellar peduncle mean FA	95.376	0.068
RSN (no significant predictor)		—

*Note:* The analysis was performed only for the 100 MS patients with assessed SDMT.

Abbreviations: FA, fractional anisotropy; GM, gray matter; LV, lesion volume; RSN, resting state networks; WM, white matter.

(i) EDSS was best explained (adjusted *R* ^2^ = 0.51, *p* < 0.001) by cingular WM lesions; FA decreases in fornix, posterior thalamic radiation, and splenium of corpus callosum; cortical, subcortical, and cerebellar GM atrophy; increased connectivity within the visual network; and age.

(ii) SDMT was best explained (adjusted *R* ^2^ = 0.49, *p* < 0.001) by periventricular (i.e., central and posterior ventricular areas) WM lesions; FA decreased around the sagittal striatum; cortical and cerebellar GM atrophy; increased connectivity within the temporal‐parietal network; and age (Table 3).

Table 4 summarizes the results of the post hoc multivariate stepwise regression for EDSS (adjusted *R* ^2^ ranges from 0.12 to 0.33, *p* < 0.001) and SDMT (adjusted *R* ^2^ ranges from 0.15 to 0.25, *p* < 0.001) using single or combined predictors from traditional MRI measures, which all show worse performance than those that use the predictors from the linked structural and functional sub‐networks (Performance scores: linked structural and functional sub‐networks: 100% versus traditional MRI derived measures: 0%. When two tests are compared, arbitrarily this metric assigns 100% to the best among the two models). Please see more details in Supporting Information.

## 4 Discussion

This study primarily aimed to assess whether integrating multimodal network patterns could enhance predictive accuracy for clinical status compared to unimodal patterns. We employed multimodal imaging data‐driven models and observed that a joint brain structural and functional network differentiates MS patients from HC. Within this network, we identified 8 sub‐networks from all modalities, exhibiting partially independent structural and functional evolution. These linked structural and functional measures improved the explanation of physical and cognitive disability compared to unimodal patterns. These findings imply the clinical relevance of a distinct and potentially overlapping pathological pattern of MS brain alterations across multiple MRI modalities.

### 4.1 A Co‐Fluctuating Pattern of Cross‐Modal Pathological Changes

Linked ICA analysis identified only one out of twenty multimodal brain networks exhibiting a difference between MS patients and the HC group. This implies that affected brain regions, mainly in areas typically involved in MS pathology (see Figure 1), are losing their architectural organization, forming a pathologically co‐fluctuating pattern specific to MS. Interestingly, most abnormal regions were close to CSF: macrostructural WM lesions, microstructural WM alterations, as well as cortical and subcortical GM atrophy were all near the ventricular spaces and the GM/CSF interface [18]. These findings could underscore the significance of soluble factors in MS, which remain understudied especially within the context of MRI. They may permeate from CSF into adjacent regions, potentially leading to diffuse damage observed in these regions of the MS brain [18].

Additionally, our linked ICA analysis showed overlapping regions between GM atrophy and FC alterations, in particular with the temporal parietal and subcortical networks (see Figure 1). This spatial overlap suggests a specific relationship between cortical macrostructural damage and related FC in MS, as previously suggested [4, 19, 20]. Finally, using GT to further categorize the abnormal brain network identified by linked ICA, we found 8 brain sub‐networks, each including different MRI modalities and formed by spatially interconnected clusters. We observed that damage in a WM region correlated with damage in the corresponding area on the brain's opposite side, regardless of the assessment modality. This observation supports the idea that axonal dysfunction manifesting remotely from focal areas of cerebral demyelination, which could drive the extensive functional and diffuse structural changes in MS despite focal WM pathology [21].

### 4.2 Multimodal (Hub) Variables Explain Physical and Cognitive Disability Better Than Unimodal Measures of Damage

The linked structural and functional sub‐networks exhibited notably greater explanatory power for disability and cognition compared to traditional MRI measures. Focal lesions in the right cingulum were identified as important for motor dysfunction, possibly due to connections with motor cortices [22]. Decreased FA suggests impaired structural integrity and axonal coherence [22, 23, 24], and was important in key areas like the fornix, thalamic radiation, and callosal splenium, potentially indicating disrupted motor functions and motor network communication [24]. Additionally, cortical, subcortical, and cerebellar GM atrophy were implicated, previously also linked to physical disability in MS [25]. Enhanced connectivity within the visual network was also linked to worse disability, which may be due to a loss of inhibition due to a loss of normal cortical architecture and a loss of thalamocortical tracts [3].

Regarding cognition, mechanisms are partially similar to motor dysfunction, as damage such as lesions and reduced FA in critical WM tracts, including the posterior thalamic radiation, genu of the corpus callosum, and anterior corona radiata may disrupt neural signal transmission across brain regions [26], leading to impaired information processing and poorer SDMT performance. Concurrently, atrophy in frontal, parietal, and temporal lobes encompasses key cognitive networks such as the frontoparietal, default‐mode, and limbic systems [27, 28]. In addition, cerebellar GM atrophy has recently been identified to also be crucial for cognition [29]. The observed increase in FC related to cognitive impairment did not overlap with those related to motor impairment, suggesting non‐overlapping functional mechanisms. Cognitively related FC increases were most important within the temporal parietal network, which overlaps with previous findings suggesting that default‐mode dysfunction is especially relevant for cognition in MS [3, 4].

### 4.3 GT Reveals a Spatially Dispersed Organization Stratified Into Clinically Relevant Brain Sub‐Networks

Looking at motor dysfunction, our GT results primarily identified the relevance of connector hubs, regions that connect different network modules together. Regionally, this included microstructural damage of the left posterior thalamic radiation and atrophy of the left thalamus in explaining physical disability, confirming previous pattern‐based analyses identifying the crucial role of the thalamus for motor dysfunction in MS [30]. This could potentially be attributed to hub overload [3, 8], leading to accelerated neurodegeneration and, consequently, more rapid clinical progression.

Conversely, lower SDMT scores were primarily correlated with the individual atlas‐stratified modality loading identified as “peripheral” (i.e., low centrality or hubness) within each subnetwork. These “peripheral” coefficients are those that are somewhat isolated, having weak correlations with damage to regions from other sub‐networks and demonstrating poor connectivity with other nodes in their community. This finding suggests that impairment of information processing speed in MS may be driven by concurrent damage to focal areas (i.e., lesions) that lack strong interconnections with each other. This observation aligns with previous work indicating that cognitive impairment was linked to altered connectivity of peripheral areas with hub areas, rather than between hub structures [31, 32].

This study has several methodological strengths. First, linked ICA used information from all MRI modalities to perform an unsupervised identification of brain regions that can differentiate MS brains from those of HC. GT retained only the altered regions that were previously detected, strongly reducing the arbitrariness of GT analysis in identifying the nodes that were subsequently clustered into the 8 sub‐networks. In addition, we combined the most commonly used MRI modalities to provide joint information about structural and functional alterations occurring in MS brains, thus providing a more integrated picture of the changes in MS brains. A final strength of the study was that it assessed the independent contribution to the changes in WM of MS brains made by focal damage, measured indirectly as the decrease in PVE of WM within lesions, and diffuse axonal damage, measured by the FA obtained from DTI. Indeed, these two processes, although concomitant, are only partially related [33] and reflect different pathological processes [34].

The main limitation of this study lies in the relatively small sample size with limited clinical evaluations and in its cross‐sectional design. It should be noted that given our limited sample size and reliance on one center with limited clinical evaluations and research‐quality scans, generalizability cannot be assessed before further validation of our findings. Additionally, the retrospective nature of this study did not allow for strict age matching between healthy controls and MS patients. While age was included as a covariate in group comparisons, some residual confounding cannot be entirely excluded. Larger MS datasets are needed to further validate the results reported here. Further research is also needed to identify which GT parameters are most informative, as the current study focused on hierarchical information using hub metrics like the participation coefficient. As such, this could lead to a loss of information on non‐hub structures, which might play an important role as well. A longitudinal study aiming at providing more comprehensive insights into the changes in brain abnormalities over time is currently ongoing.

In conclusion, the present study used linked‐ICA to identify a clinically relevant multimodal brain pattern characterized by co‐fluctuating focal and diffuse alterations in specific WM regions, regional GM atrophy, and functional network changes. Furthermore, GT analysis identified the key regions within each modality that drove these cross‐modal relations, highlighting the value of more advanced network processing of multimodal data in MS. Finally, this multimodal approach enhanced the explanation of clinical status beyond unimodal variables, underscoring the clinical relevance of a network approach to understand complex symptoms and clinical progression in MS.

## Conflicts of Interest

J.Z., M.L.S., M.M., L.L., G.G. have nothing to disclose. M.B. is co‐founder of Siena Imaging s.r.l. R.C. received speaker honoraria/travel support from Roche, Merck Serono, UCB, Sanofi‐Genzyme, Novartis, and Janssen, received research grant from the Italian Ministry of University and Research. E.P. received compensation for travel grants, participation in advisory board and/or speaking activities from Biogen, Merck Serono, Sanofi, Teva and Novartis; and serves on the editorial board of Frontiers in Neurology and Brain Sciences. M.P.A. served on scientific advisory boards for and has received speaker honoraria and research support from Biogen Idec, Merck Serono, Bayer Schering Pharma and Sanofi‐Aventis, and serves on the editorial board of Multiple Sclerosis Journal and BMC Neurology. M.M.S. serves on the editorial board of Neurology and Frontiers in Neurology and Multiple Sclerosis Journal, receives research support from the Dutch MS Research Foundation, Eurostars‐EUREKA, ARSEP, Amsterdam Neuroscience and ZonMW and has served as a consultant for or received research support from EIP Pharma, Atara Biotherapeutics, Biogen, Celgene/Bristol Meyers Squibb, Genzyme, MedDay and Merck. N.D.S. has received honoraria from Biogen‐Idec, Bristol Myers Squibb, Celgene, Genzyme, Immunic, Merck Serono, Novartis, Roche and Teva for consulting services, speaking, and travel support. He serves on advisory boards for Merck, Novartis, Biogen‐Idec, Roche, and Genzyme, Immunic. He has received research grant support from the Italian MS Society and is co‐founder of Siena Imaging s.r.l.

## Supporting information


**Appendix S1:** ene70367‐sup‐0001‐AppendixS1.docx.
**Figure S1:** Main steps of creating white matter lesion map. WM: white matter; PVE: partial volume effect; LPM: lesion probability mapping.
**Figure S2:** Comparison of different regression models for EDSS. The indices were uniformly rescaled to span a range from 0 to 1. Therefore, for *R* ^2^, *R* ^2^ adjusted, AIC_wt, AICc_wt and BIC_wt, data points that are closer to the center indicate poorer fit indices. Conversely, for RMSE and Sigma, data points that are further from the center indicate poorer fit indices. EDSS: Expanded Disability Status Scale; model1: linked structural and functional sub‐networks; model2: combination of traditional MRI measures; RMSE: root mean square error; AIC_wt: weight of Akaike's Information Criterion; AICc_wt: weight of Akaike's Information Criterion corrected; BIC_wt: weight of Bayesian Information Criterion.
**Figure S3:** Comparison of different regression models for SDMT. The indices were uniformly rescaled to span a range from 0 to 1. Therefore, for *R* ^2^, *R* ^2^ adjusted, AIC_wt, AICc_wt and BIC_wt, data points that are closer to the center indicate poorer fit indices. Conversely, for RMSE and Sigma, data points that are further from the center indicate poorer fit indices. SDMT: Symbol Digit Modalities Test; model1: linked structural and functional sub‐networks; model2: combination of traditional MRI measures; RMSE: root mean square error; AIC_wt: weight of Akaike's Information Criterion; AICc_wt: weight of Akaike's Information Criterion corrected; BIC_wt: weight of Bayesian Information Criterion.
**Table S1:** List of Traditional MRI Measures Used for the Initial Stepwise Regression Models.
**Table S2:** List of the Changed Multimodal Hierarchical Networks with Their Corresponding Involved Brain Regions in Multiple Sclerosis.
**Table S3:** Results from the Performance Comparison of Different Regression Models for EDSS.
**Table S4:** Results from the Performance Comparison of Different Regression Models for SDMT.

## Data Availability

Research data are not shared.
