# Supplementary material for: Clinically Relevant Patterns of Co‐Fluctuating Structure and Function in Multiple Sclerosis
Source: Eur J Neurol. 2025 Sep 29;32(10):e70367. doi: 10.1111/ene.70367 (PMC12477496; doi:10.1111/ene.70367)
Supplement: Supplementary file 1 — Appendix S1: ene70367‐sup‐0001‐AppendixS1.docx. Figure S1: Main steps of creating white matter lesion map. WM: white matter; PVE: partial volume effect; LPM: lesion probability mapping. Figure S2: Comparison of different regression models for EDSS. The indices were uniformly rescaled to span a range from 0 to 1. Therefore, for R 2, R 2 adjusted, AIC_wt, AICc_wt and BIC_wt, data points that are closer to the center indicate poorer fit indices. Conversely, for RMSE and Sigma, data points that are further from the center indicate poorer fit indices. EDSS: Expanded Disability Status Scale; model1: linked structural and functional sub‐networks; model2: combination of traditional MRI measures; RMSE: root mean square error; AIC_wt: weight of Akaike's Information Criterion; AICc_wt: weight of Akaike's Information Criterion corrected; BIC_wt: weight of Bayesian Information Criterion. Figure S3: Comparison of different regression models for SDMT. The indices were uniformly rescaled to span a range from 0 to 1. Therefore, for R 2, R 2 adjusted, AIC_wt, AICc_wt and BIC_wt, data points that are closer to the center indicate poorer fit indices. Conversely, for RMSE and Sigma, data points that are further from the center indicate poorer fit indices. SDMT: Symbol Digit Modalities Test; model1: linked structural and functional sub‐networks; model2: combination of traditional MRI measures; RMSE: root mean square error; AIC_wt: weight of Akaike's Information Criterion; AICc_wt: weight of Akaike's Information Criterion corrected; BIC_wt: weight of Bayesian Information Criterion. Table S1: List of Traditional MRI Measures Used for the Initial Stepwise Regression Models. Table S2: List of the Changed Multimodal Hierarchical Networks with Their Corresponding Involved Brain Regions in Multiple Sclerosis. Table S3: Results from the Performance Comparison of Different Regression Models for EDSS. Table S4: Results from the Performance Comparison of Different Regression Models for SDMT. [file ENE-32-e70367-s001.docx]

**Supplementary** **Material**

**MRI Data Acquisition**

All participants were scanned on a 3T MRI scanner (Philips Medical Systems, Best, The Netherlands) located at Meyer University Hospital, Florence, with the following MRI protocol: (1) dual-echo, turbo spin-echo sequence: repetition time (TR)/echo time (TE)1/TE2 = 4000/10/100 ms, flip angle = 90°, voxel size = 1×1×3 mm, 44 slices to acquire proton density (PD) and T2-weighted (T2-W) images; (2) 2-dimensional (2D) ﬂuid-attenuated inversion recovery (FLAIR) sequence: TR/TE = 11000/125 ms, flip angle variable, voxel size = 1×1×3 mm, 44 slices; or 3-dimensional (3D) FLAIR sequence: TR/TE = 8000/125 ms, flip angle variable, voxel size = 1 mm^3^, 175 sagittal slices; (3) DTI sequence: TR = 7036 ms; TE = 96 ms, flip angle = 90°, voxel size = 2.5 mm^3^, 50 slices, diffusion sampled on the half sphere with 32 diffusion directions and b-value = 900 s/mm^2^; (4) T2*-weighted single-shot echo planar imaging sequence for resting functional MRI (FMRI): 200 volumes, TR = 3000 ms, TE = 35 ms, flip angle = 90°, voxel size = 1.87×1.87×4 mm, 30 slices; and (5) 3D T1-weighted (T1-W) sequence: TR = 10 ms, TE = 4 ms, flip angle = 8°, voxel size = 1 mm^3^, 192 slices.

**Creation of Intensity Normalized 4D Image**

**White Matter Lesions**

Since the lesion masks are binary (intensity of 1 for lesion voxels and 0 for healthy voxels), they cannot be used directly in ICA. Thus, we implemented a new approach to obtain an individual map of continuous values reflecting focal WM damage.

1. A lesion probability map (LPM), used here as a common spatial brain mask, was created using FSL (FMRIB Software Library, http://www.fmrib.ox.ac.uk/fsl). The LPM was generated by averaging the lesion masks, for each voxel, non-linearly registered to standard space (MNI152 brain) using the nonlinear registration tool FNIRT^1–3^. Each lesion mask was outlined using BIANCA-MS, a fully-automated supervised method for detecting WMH hyperintensity^4^, using T1-W together with PD or FLAIR. Each lesion map was then reviewed by an expert evaluator (RTB), blinded to patient identity, using a semiautomated local threshold technique (Jim 6.0, Xinapse System, Leicester, UK). Total lesion volume (LV in mm^3^) was also calculated by multiplying the lesion area (in mm^2^) by the slice thickness (in mm).
2. WM focal destructive damage is reflected as hypo-intensities on T1-W images and related to the decrease of WM partial volume estimation (PVE), thus for each subject we retained the WM-PVE within the LPM previously obtained. The spatial transformation to MNI space was computed by registering the lesion-filled 3D T1-W image to the MNI template using the nonlinear registration tool FNIRT^1^, following N4 bias field correction. Lesion filling was applied at this stage to minimize distortion of the transformation caused by periventricular lesion burden and to improve template alignment. The non–lesion-filled WM-PVE maps, obtained by segmentation with SIENAX 2.0^5,6^ were then transformed into the MNI standard space using the spatial transformation obtained from the lesion-filled images. Then, for each subject, only values in the voxels within the LPM were retained. These masked WM-PVE images were multiplied by -1 to make the visual interpretation of the map more intuitive, then smoothed with a Gaussian smoothing kernel of 2 mm sigma, and these final values then represented the WM lesion images used as inputs for ICA. These steps are illustrated in Supplementary Fig. 1.

**White Matter Microstructure**

DTI data were preprocessed using the FSL program *eddy*, which corrects for eddy current-induced distortions, subject movement and signal dropout^7^. After this we used FDT (FMRIB Diffusion Toolbox) to obtain fractional anisotropy (FA) images by ﬁtting a diffusion tensor model at each voxel. The FA image from each subject was registered to a standard-space image (FMRIB58_FA) using nonlinear registration tool FNIRT^1^, which were then smoothed with a 2 mm sigma Gaussian kernel^8^. An FA mask, which was generated from the group averaged FA maps (thresholded at FA values > 0.2) in standard space was employed to include only WM voxels^9^.

**Brain Volumes**

Lesion-ﬁlled^2^ 3D T1-W images were used to calculate normalized brain volume (NBV), normalized GM volume (NGMV), normalized peripheral GM volume (NPGMV), and normalized subcortical GM volume (NSGMV) with SIENAX 2.0^5,6^.

**Grey Matter Voxel-based Morphometry**

FSL voxel based morphometry (VBM, <https://fsl.fmrib.ox.ac.uk/fsl/fslwiki/FSLVBM>), is an optimized voxel-based morphometry analysis protocol that was performed on 3D T1-weighted images after “filling” hypointense lesions with intensities of the surrounding normal-appearing WM, in order to improve registration and segmentation and thus the resultant volumetric assessment^2^. First, T1-weighted images were brain-extracted and the GM segmented with SIENAX 2.0^5,6^. A left–right symmetric, study-specific template^10,11^ was created by: (1) registering a balanced sample (26 MS patients and 26 HC) of brain-extracted, GM-segmented images to the MNI 152 standard space “avg152T1_gray” template using nonlinear registration^12^ and (2) averaging these images and flipped versions of these (with x-axis flipping)^10,13^. After nonlinearly normalizing all the GM images to this symmetric, study-specific GM template, the modulated GM images were smoothed with an isotropic Gaussian kernel with a sigma of 2 mm^11,13^.

**Functional Network**

The first five time-points of resting FMRI data were removed to allow signal stability, followed by realignment of the remaining volumes to the middle volume (head motion correction), grand mean scaling, and spatial smoothing with a Gaussian kernel of 6 mm FWHM. ICA-AROMA (independent component analysis-based automatic removal of motion artifacts) was then applied to minimize motion-related artifacts^14^. Average signals extracted from WM and cerebrospinal fluid (CSF) were regressed out to eliminate residual structured noise, and then high-pass temporal filtering (cut-off frequency = 100 s) was applied. Then, images were registered to the MNI152 standard brain, using the T1-W image as an intermediate, using FNIRT. Group ICA using MELODIC (multivariate exploratory linear optimized decomposition into independent components [ICs]) was conducted on concatenated, normalized images, which were decomposed into 25 ICs, based on commonly studied resting state networks (RSNs)^15,16^. Visual inspection, frequency analysis, and template-matching were used to select 11 networks^17,18^. Corresponding subject-speciﬁc network maps were then generated by the dual-regression procedure^19^.

**Preprocessing Steps for Linked ICA**

To perform the linked ICA, two preliminary steps were performed:

1. Each modality was preprocessed to obtain the 4D MRI maps usually created for a single voxel-wise modality (e.g., using FSL-VBM, for the 3D T1-W images, to obtain the modulated partial volume effect (PVE) of each subject’s GM map in standard space). This preliminary step addresses two conditions that must be met for each modality:
   1. given a modality, the identification of a spatial brain mask shared among all study subjects, uniquely indicating all voxels to be processed for that single modality (e.g., the fractional anisotropy (FA) obtained for diffusion tensor imaging (DTI)) and,
   2. within this mask, an individual map with continuous values for each subject and with intensities that can reflect changes in biological processes (e.g., a lower GM PVE in the cortex reflects greater atrophy).
2. An overall intensity normalization of all 4D images.

**Intensity Normalization**

An overall intensity normalization of all 4D images was performed to ensure that all modalities had the same range of values. To do this, spatial maps of WM lesions, FA, GM VBM and RSN were individually reshaped into one-dimensional vectors. These vectors were then stacked, forming a matrix (N_subj_ × N_voxel_) for each of the four modalities. These matrices were subsequently normalized to achieve the same average sum of squares (calculated across all subjects and all voxels for each modality), ensuring that all modalities had consistent ranges^20^.

**Linked ICA Analysis**

Each IC comprises a group-level spatial map for WM lesions, FA, GM VBM and RSN respectively, and a participant-specific IC loading coefficient (i.e., the degree to which a pattern is present at an individual participant level) across WM lesions, FA, GM VBM and RSN, representing the contribution that an IC makes to the participant’s data. Only those components showing a different loading in MS patients compared to HC were explored further using GT. Since structural and functional modalities are known to be related in the normal human brain, this selection procedure was chosen to solely focus on pathological components driven by MS.

We first identified ICs that exhibited differences between MS and HC (please refer to the 'Statistical analysis' section for more in-depth information). For each modality, the spatial map of the identified ICs was subjected to a threshold of |Z| > 2. A voxel with a higher |Z| value for a specific modality indicates greater variation of that modality at that voxel. Through a rigorous intersection calculation between each modality and their corresponding atlases, we successfully pinpointed brain regions that exhibited consistent involvement within each modality. These identified brain regions are now being considered as potential nodes in graph theory (neuromarkers): Hence, in the case of the identified ICs displaying differences between MS and HC, only the voxels within regions or specific portions of regions defined by the atlases were retained and utilized for calculating node loadings:

1. The intersection between the spatial map of WM lesions and the ICBM-DTI-81 atlases.

2. The intersection between the spatial map of FA and the ICBM-DTI-81 atlases.

3. The intersection between the spatial map of GM VBM and the Harvard-Oxford cortical, subcortical structural atlases, along with the Probabilistic cerebellar atlas.

4. The intersection between the spatial maps of RSN and the Smith-10 ICA-based network parcellation atlas, enhanced by the complementary Yeo-17 network atlas and an additional subcortical network not present in the original atlas.

Subsequently, we computed subject-specific averaged loading coefficients for each region and modality. A multivariate regression procedure using the atlas-stratified modality-IC spatial maps was implemented to create the loading coefficients:

1. For each modality, we performed multivariate regression of the atlas-stratified IC spatial maps against the original data (i.e., the normalized WM lesions, FA, GM VBM, and RSN spatial maps) to obtain the IC-weighted individual modality-driven spatial maps^21^.

2. The mean of the voxels within the regressed spatial map was computed for each subject, resulting in a N_subj_ × 1 feature vector for each modality. The subject-specific averaged loading coefficients were utilized as node weights.

**Control Analysis**

As a control analysis, we repeated the linked ICA after excluding the 10 progressive MS patients (3 SPMS and 7 PPMS). The identified components and subsequent graph measures showed minimal differences, with largely overlapping spatial patterns and comparable subject loadings. This suggests that including different clinical phenotypes did not substantially affect the main findings.

**Calculation of Graph Theory Indices**

These node weights were used to create a partial correlation matrix using Gaussian graphical models (GGM) ^22,23^ with the values of partial correlation used to create the edges of our GT. The partial correlation matrix was converted to a binary network binarizing all the significant (*P* < 0.05) partial correlations obtained with the GGM approach and without any a-priori threshold selection. The Louvain community detection method^24^ (https://networkx.github.io/) was conducted on the binary network to reveal the structure of the network. Louvain method heuristically maximize the quotient graph Q, a measure of the community strength varying from 0 (random partition in community) to 1 and usually considered acceptable with values from 0.3 to 0.7)^25^.

The modularity *Q* is a measure of the structure of networks, or graphs, which measures the strength of division of a network into communities^26^. *Q* is written analytically as follows:

$$Q=\frac{1}{2m}\sum_{ij} \left( A_{ij}-\frac{k_{i}k_{j}}{2m} \right)\delta\left( c_{i},c_{j} \right)$$

where the network is represented by an adjacency matrix *A* with n nodes and *m* edges, and *A_ij_* = 0 means there is no edge between nodes *i* and *j* and *A_ij_* = 1 means there is an edge between the two. The degrees of nodes *i* and *j* are represented by *k_i_* and *k_j_* respectively. The degree of a node is defined as the summation of all connections between that particular node and other nodes. *c_i_* is the community to which node *i* belongs. In network science, a community refers to a subset of nodes within a network that are densely interconnected among themselves compared to the rest of the network. Finally, *δ(α, β)* =*1* if *α* = *β* or *δ(α, β) = 0* if *α ≠ β*.

For a specific community assignment, the participation coefficient (*PC*)^27^ of node *i* is defined as:

$${PC}_{i}=1-\sum_{s=1}^{N_{M}} \left( \frac{k_{is}}{k_{i}} \right)^{2}$$

where *N_M_* is the total number of communities, *k_is_* is the number of edges of node *i* to nodes in community *s*, and *k_i_* is the total degree of node *i*.

We also computed the within-community (*WC*) ^27^ strength value for node *i* as follows:

$${WC}_{i}=\frac{k_{i}-\bar{k}_{is}}{\sigma_{ks}}$$

where *k_i_* is the degree of node *i*, $\bar{k}_{is}$ is the average of *k* over all of the nodes in community *s*, and $\sigma_{ks}$ is the standard deviation of the degree *k* in community *s.* Thus, the *WC* strength measures how well-connected node *i* is to other nodes in its community relative to all other nodes in this community.

**Performance Comparison of Different Regression Models**

For a quantitative comparison of the best selected models – one based on regional and modality-specific average loading coefficients and the other using a combination of traditional MRI measures – we computed a performance score for each model^28,29^. The performance score was derived using multiple information criteria, including R^2^, adjusted R^2^, root-mean-square error (RMSE), sigma, Akaike information criterion (AIC) weights, corrected Akaike's Information Criterion (AICc) weights, and Bayesian information criterion (BIC) weights^28,29^. The calculation process entails normalizing all indices by rescaling them to a range from 0 to 1. Subsequently, the performance score for each model is determined by calculating the mean value of all indices. When comparing the performance score between two models, the winning one will have the higher performance score, compared to the losing model^29^. The results are shown in Supplementary Fig. 2-3 and Supplementary Table 3-4.

**
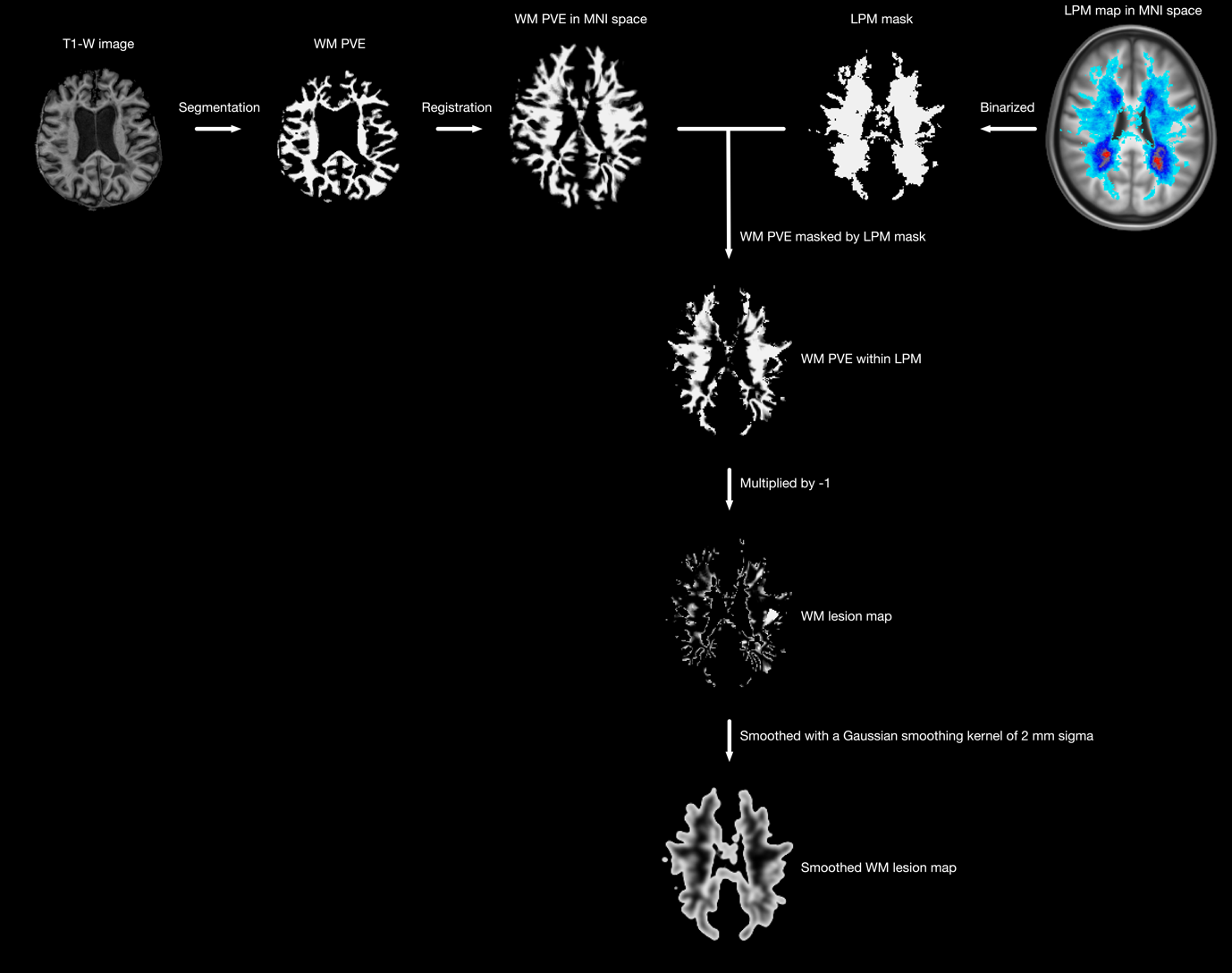
**

**Supplementary Figure 1: Main steps of creating white matter lesion map.** WM: white matter; PVE: partial volume effect; LPM: lesion probability mapping.

**
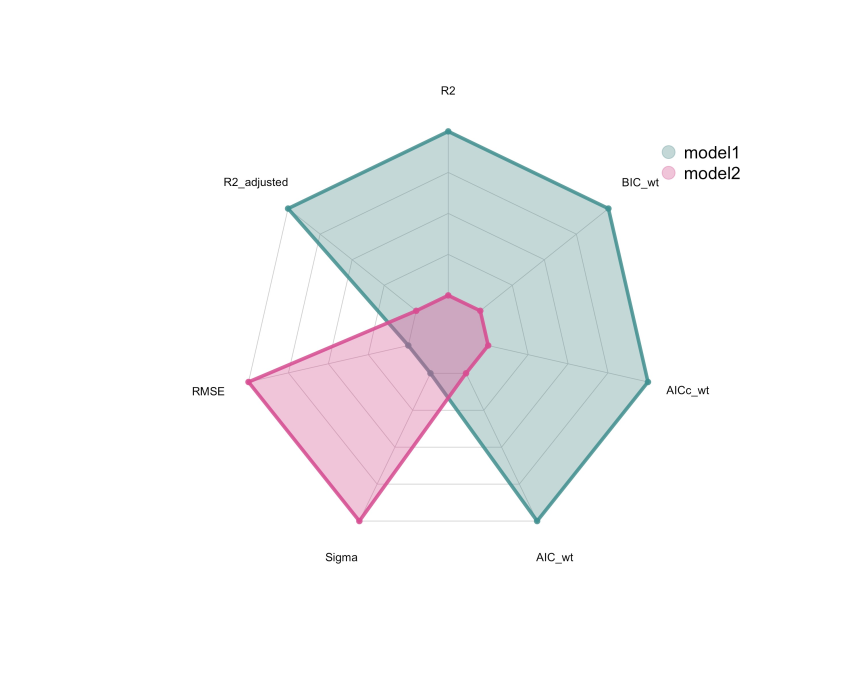
**

**Supplementary Figure 2: Comparison of different regression models for EDSS.** The indices were uniformly rescaled to span a range from 0 to 1. Therefore, for R^2^, R^2^ adjusted, AIC_wt, AICc_wt and BIC_wt, data points that are closer to the center indicate poorer fit indices. Conversely, for RMSE and Sigma, data points that are further from the center indicate poorer fit indices. EDSS: Expanded Disability Status Scale; model1: linked structural and functional sub-networks; model2: combination of traditional MRI measures; RMSE: root mean square error; AIC_wt: weight of Akaike's Information Criterion; AICc_wt: weight of Akaike's Information Criterion corrected; BIC_wt: weight of Bayesian Information Criterion.

**
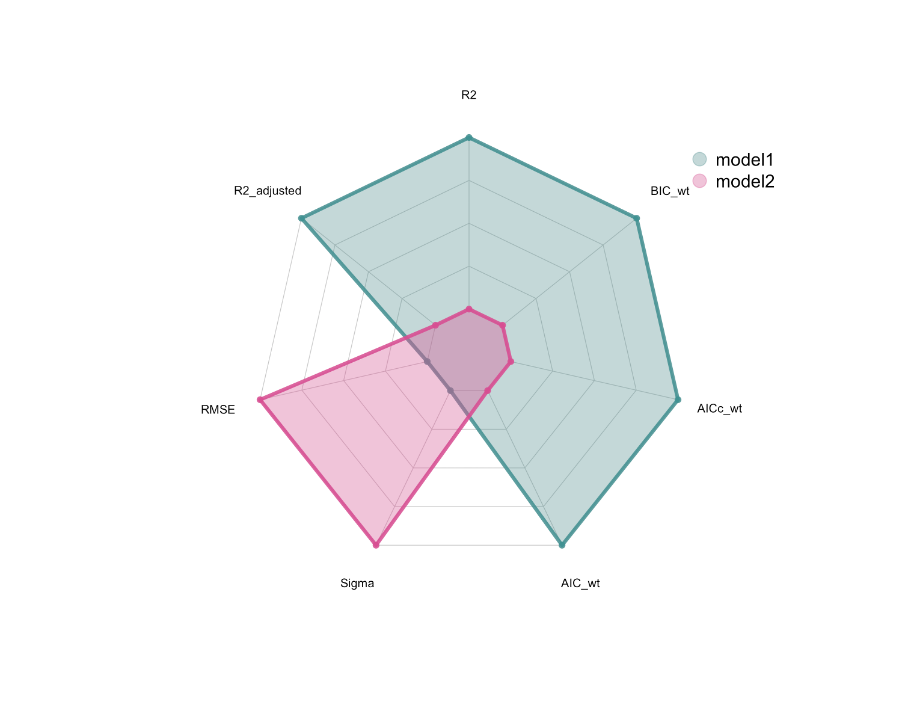
Supplementary Figure 3: Comparison of different regression models for SDMT.** The indices were uniformly rescaled to span a range from 0 to 1. Therefore, for R^2^, R^2^ adjusted, AIC_wt, AICc_wt and BIC_wt, data points that are closer to the center indicate poorer fit indices. Conversely, for RMSE and Sigma, data points that are further from the center indicate poorer fit indices. SDMT: Symbol Digit Modalities Test; model1: linked structural and functional sub-networks; model2: combination of traditional MRI measures; RMSE: root mean square error; AIC_wt: weight of Akaike's Information Criterion; AICc_wt: weight of Akaike's Information Criterion corrected; BIC_wt: weight of Bayesian Information Criterion.

**Supplementary Table 1** **List of Traditional MRI Measures Used for the Initial Stepwise Regression Models**

| **MRI measure** | **Region** |
| --- | --- |
| **LV** | Whole brain |
|  |  |
| **Brain volume** | NBV |
|  | NGMV |
|  | NSGMV |
|  |  |
| **Mean FA value** | Middle cerebellar peduncle |
|  | Pontine crossing tract |
|  | Genu of corpus callosum |
|  | Body of corpus callosum |
|  | Splenium of corpus callosum |
|  | Fornix (column and body of fornix) |
|  | Corticospinal tract R |
|  | Corticospinal tract L |
|  | Medial lemniscus R |
|  | Medial lemniscus L |
|  | Inferior cerebellar peduncle R |
|  | Inferior cerebellar peduncle L |
|  | Superior cerebellar peduncle R |
|  | Superior cerebellar peduncle L |
|  | Cerebral peduncle R |
|  | Cerebral peduncle L |
|  | Anterior limb of internal capsule R |
|  | Anterior limb of internal capsule L |
|  | Posterior limb of internal capsule R |
|  | Posterior limb of internal capsule L |
|  | Retrolenticular part of internal capsule R |
|  | Retrolenticular part of internal capsule L |
|  | Anterior corona radiata R |
|  | Anterior corona radiata L |
|  | Superior corona radiata R |
|  | Superior corona radiata L |
|  | Posterior corona radiata R |
|  | Posterior corona radiata L |
|  | Posterior thalamic radiation R |
|  | Posterior thalamic radiation L |
|  | Sagittal stratum R |
|  | Sagittal stratum L |
|  | External capsule R |
|  | External capsule L |
|  | Cingulum (cingulate gyrus) R |
|  | Cingulum (cingulate gyrus) L |
|  | Cingulum (hippocampus) R |
|  | Cingulum (hippocampus) L |
|  | Fornix (cres) / Stria terminalis R |
|  | Fornix (cres) / Stria terminalis L |
|  | Superior longitudinal fasciculus R |
|  | Superior longitudinal fasciculus L |
|  | Superior fronto-occipital fasciculus R |
|  | Superior fronto-occipital fasciculus L |
|  | Uncinate fasciculus R |
|  | Uncinate fasciculus L |
|  | Tapetum R |
|  | Tapetum L |
|  | Middle cerebellar peduncle |
|  |  |
| **Functional network connectivity** | Visual network (medial) |
|  | Default mode network |
|  | Visual network (lateral) |
|  | Executive control network |
|  | Visual network (occipital pole) |
|  | Temporal parietal network |
|  | Sensorimotor network |
|  | Left frontoparietal network |
|  | Right frontoparietal network |
|  | Cerebellar network |
|  | Subcortical network |

Abbreviations

LV: lesion volume; NBV: normalized brain volume, NGMV: normalized grey matte volume; NPGMV: normalized peripheral grey matter volume; NSGMV: normalized subcortical grey matter volume; R: right; L: left.

**Supplementary Table 2** **List of the Changed Multimodal Hierarchical Networks with Their Corresponding Involved Brain Regions in Multiple Sclerosis**

| **Network** | **Regions** |
| --- | --- |
| **Central WM lesion mediated network** | Right crus II cerebellum (GM, ↓), Right cerebral peduncle (lesion, ↑), Brain-Stem (GM, ↓), Right precentral gyrus (GM, ↓), Visual network (medial, ↑), Body of corpus callosum (lesion, ↑), Right posterior limb of internal capsule (lesion, ↑), Body of corpus callosum (FA, ↓), Splenium of corpus callosum (FA, ↓), Cerebellar network (↑) |
|  |  |
| **Left anterior ventricular WM microstructural damage mediated network** | Right cingulum (lesion, ↑), Right juxtapositional lobule (GM, ↓), Left IX cerebellum (GM, ↓), Left superior corona radiata (FA, ↓), Left VIIIb cerebellum (GM, ↓), Right postcentral gyrus (GM, ↓), Left VIIIa cerebellum (GM, ↓), Right IX cerebellum (GM, ↓), Left postcentral gyrus (GM, ↓), Right supramarginal gyrus (GM, ↓), Left juxtapositional lobule (GM, ↓), Left anterior corona radiata (FA, ↓), Left frontoparietal network (↑), Right VIIIb cerebellum (GM, ↓), Right superior frontal gyrus (GM, ↓), Right superior parietal lobule (GM, ↓), Visual network (lateral, ↑), Right angular gyrus (GM, ↓) |
|  |  |
| **L****eft posterior ventricular WM microstructural damage mediated network** | Left ventral striatum (GM, ↓), Right tapetum (lesion, ↑), Right posterior corona radiata (lesion, ↑), Left tapetum (lesion, ↑), Right posterior thalamic radiation (lesion, ↑), Right insula cortex (GM, ↓), Right superior longitudinal fasciculus (lesion, ↑), Right Heschl’s gyrus (GM, ↓), Left anterior corona radiata (lesion, ↑), Left posterior thalamic radiation (FA, ↓), Right sagittal stratum (lesion, ↑), Right frontal orbital cortex (GM, ↓), Right subcallosal cortex (GM, ↓), Left frontal orbital cortex (GM, ↓), Right superior corona radiata (lesion, ↑), Left temporal pole (GM, ↓), Right superior longitudinal fasciculus (FA, ↓), Genu of corpus callosum (lesion, ↑) |
|  |  |
| **L****eft lateral ventricular WM microstructural damage mediated network** | Left cingulum (lesion, ↑), Right thalamus (GM, ↓), Left retrolenticular part of internal capsule (FA, ↓), Left inferior frontal gyrus (GM, ↓), Left frontal operculum cortex (GM, ↓), Right amygdala (GM, ↓), Left lateral occipital cortex (GM, ↓), Left occipital pole (GM, ↓), Left sagittal stratum (FA, ↓), Left posterior corona radiata (FA, ↓), Splenium of corpus callosum (lesion, ↑), Left tapetum (FA, ↓), Sensorimotor network (↑) |
|  |  |
| **Left subcortical GM atrophy mediated network** | Left planum temporale (GM, ↓), Left putamen (GM, ↓), Left lingual gyrus (GM, ↓), Left amygdala (GM, ↓), Left caudate (GM, ↓), Left insula (GM, ↓), Right lateral occipital cortex (GM, ↓), Left hippocampus (GM, ↓), Left thalamus (GM, ↓), Right caudate (GM, ↓), Right middle temporal gyrus (GM, ↓), Left subcallosal cortex (GM, ↓), Right ventral striatum (GM, ↓), Default mode network (↑), Left Heschl’s gyrus (GM, ↓), Left Temporal occipital fusiform cortex (GM, ↓), Right putamen (GM, ↓), Right middle frontal gyrus (GM, ↓) |
|  |  |
| **Right subcortical GM atrophy mediated network** | Right posterior thalamic radiation (FA, ↓), Left posterior thalamic radiation (lesion, ↑), Right hippocampus (GM, ↓), Left lateral occipital cortex (GM, ↓), Left precuneous cortex (GM, ↓), Left sagittal stratum (lesion, ↑), Left angular gyrus (GM, ↓), Right retrolenticular part of internal capsule (lesion, ↑), Right fornix (FA, ↓), Right sagittal stratum (FA, ↓), Right retrolenticular part of internal capsule (FA, ↓), Left fornix (FA, ↓), Right posterior corona radiata (FA, ↓), Left supramarginal gyrus (GM, ↓), Right superior corona radiata (FA, ↓), Left posterior corona radiata (lesion, ↑), Right tapetum (FA, ↓), Left superior corona radiata (lesion, ↑), Right crus I cerebellum (GM, ↓) |
|  |  |
| **Left cerebellar atrophy mediated network** | Subcortical network (↑), Fornix (column and body, FA, ↓), Left cuneal cortex (GM, ↓), Left supramarginal gyrus (GM, ↓), Left parietal operculum cortex (GM, ↓), Left crus II cerebellum (GM, ↓), Right anterior corona radiata (FA, ↓), Right occipital pole (GM, ↓), Left crus I cerebellum (GM, ↓), Left superior parietal lobule (GM, ↓) |
| **Temporal RSN activity alteration mediated network** | Left precentral gyrus (GM, ↓), Right frontoparietal network (↑), Right lingual gyrus (GM, ↓), Vermis IX cerebellum (GM, ↓), Right lateral occipital cortex (GM, ↓), Left superior frontal gyrus (GM, ↓), Temporal parietal network (↑), Left occipital fusiform gyrus (GM, ↓), Right occipital fusiform gyrus (GM, ↓), Left superior longitudinal fasciculus (FA, ↓), Left superior longitudinal fasciculus (lesion, ↑) |

Abbreviations

WM: white matter; GM: grey matter; FA: fractional anisotropy. ↑: compared to healthy controls, multiple sclerosis patients presented increased lesions and resting state network activity; ↓: compared to healthy controls, multiple sclerosis patients presented decreased grey matter volume and white matter fractional anisotropy.

**Supplementary** **Table 3** **Results from the Performance** **Comparison of Different Regression Models for EDSS**

| **Metrics** | **Model** | |
| --- | --- | --- |
|  | **Linked structural and functional sub-networks** | **Combination of traditional MRI measures** |
| Performance-Score | 100% | 0.00% |
| R^2^ | 0.578 | 0.367 |
| R^2^ (adj.) | 0.515 | 0.330 |
| RMSE | 0.933 | 1.142 |
| Sigma | 1.004 | 1.179 |
| AIC weights | 1.000 | 6.96e-09 |
| AICc weights | 1.000 | 1.25e-07 |
| BIC weights | 0.912 | 0.088 |

Abbreviations

EDSS: Expanded Disability Status Scale; ICA: independent component analysis; GT: graph theory; adj.: adjusted; RMSE: root mean square error; AIC: Akaike's Information Criterion; AICc: Akaike's Information Criterion corrected; BIC: Bayesian Information Criterion.

For the information criteria (IC, i.e., AIC, AICc, BIC), weights are computed as:

$$W=exp\left( -0.5\times delta\_IC \right)/{sum\left( exp\left( -0.5\times delta\_IC \right) \right)}$$

Where $delta\_IC$ is the difference between the model's *IC* value and the smallest *IC* value in the model set ^28^.

**Supplementary Table 4** **Results from the Performance Comparison of Different Regression Models for SDMT**

| **Metrics** | **Model** | |
| --- | --- | --- |
|  | **Linked structural and functional sub-networks** | **Combination of traditional MRI measures** |
| Performance-Score | 100% | 0.00% |
| R^2^ | 0.547 | 0.292 |
| R^2^ (adj.) | 0.478 | 0.254 |
| RMSE | 7.932 | 9.917 |
| Sigma | 8.553 | 10.228 |
| AIC weights | 1.000 | 5.94e-07 |
| AICc weights | 1.000 | 5.63e-06 |
| BIC weights | 0.980 | 0.020 |

Abbreviations

SDMT: Symbol Digit Modalities Test; ICA: independent component analysis; GT: graph theory; adj.: adjusted; RMSE: root mean square error; AIC: Akaike's Information Criterion; AICc: Akaike's Information Criterion corrected; BIC: Bayesian Information Criterion.

For the information criteria (IC, i.e., AIC, AICc, BIC), weights are computed as:

$$W=exp\left( -0.5\times delta\_IC \right)/{sum\left( exp\left( -0.5\times delta\_IC \right) \right)}$$

Where $delta\_IC$ is the difference between the model's *IC* value and the smallest *IC* value in the model set ^28^.

**References**

1. Andersson JL, Jenkinson M, Smith S, others. Non-linear registration, aka spatial normalisation. *FMRIB technical report TR07JA2*. Published online 2007. Accessed July 7, 2016. https://www.fmrib.ox.ac.uk/analysis/techrep/tr07ja2/tr07ja2.pdf

2. Battaglini M, Jenkinson M, De Stefano N. Evaluating and reducing the impact of white matter lesions on brain volume measurements. *Hum Brain Mapp*. 2012;33(9):2062-2071. doi:10.1002/hbm.21344

3. Rossi F, Giorgio A, Battaglini M, et al. Relevance of Brain Lesion Location to Cognition in Relapsing Multiple Sclerosis. *PLOS ONE*. 2012;7(11):e44826. doi:10.1371/journal.pone.0044826

4. Gentile G, Jenkinson M, Griffanti L, et al. BIANCA-MS: An optimized tool for automated multiple sclerosis lesion segmentation. *Hum Brain Mapp*. 2023;44(14):4893-4913. doi:10.1002/hbm.26424

5. Battaglini M, Jenkinson M, Stefano ND. SIENA-XL for improving the assessment of gray and white matter volume changes on brain MRI. *Human Brain Mapping*. 2018;39(3):1063-1077. doi:10.1002/hbm.23828

6. Battaglini M, Gentile G, Luchetti L, et al. Lifespan normative data on rates of brain volume changes. *Neurobiology of Aging*. 2019;81:30-37. doi:10.1016/j.neurobiolaging.2019.05.010

7. Andersson JLR, Sotiropoulos SN. An integrated approach to correction for off-resonance effects and subject movement in diffusion MR imaging. *Neuroimage*. 2016;125:1063-1078. doi:10.1016/j.neuroimage.2015.10.019

8. Fu Z, Iraji A, Caprihan A, et al. In search of multimodal brain alterations in Alzheimer’s and Binswanger’s disease. *NeuroImage: Clinical*. Published online July 15, 2019:101937. doi:10.1016/j.nicl.2019.101937

9. Mohammadi S, Keller SS, Glauche V, et al. The Influence of Spatial Registration on Detection of Cerebral Asymmetries Using Voxel-Based Statistics of Fractional Anisotropy Images and TBSS. *PLOS ONE*. 2012;7(6):e36851. doi:10.1371/journal.pone.0036851

10. Douaud G, Smith S, Jenkinson M, et al. Anatomically related grey and white matter abnormalities in adolescent-onset schizophrenia. *Brain: A Journal of Neurology*. 2007;130(Pt 9):2375-2386. doi:10.1093/brain/awm184

11. Douaud G, Groves AR, Tamnes CK, et al. A common brain network links development, aging, and vulnerability to disease. *Proc Natl Acad Sci*. 2014;111(49):17648-17653. doi:10.1073/pnas.1410378111

12. Jenkinson M, Beckmann CF, Behrens TEJ, Woolrich MW, Smith SM. FSL. *Neuroimage*. 2012;62(2):782-790. doi:10.1016/j.neuroimage.2011.09.015

13. Smith S, Duff E, Groves A, et al. Structural variability in the human brain reflects fine-grained functional architecture at the population level. *J Neurosci*. Published online May 31, 2019:2912-2918. doi:10.1523/JNEUROSCI.2912-18.2019

14. Pruim RHR, Mennes M, van Rooij D, Llera A, Buitelaar JK, Beckmann CF. ICA-AROMA: A robust ICA-based strategy for removing motion artifacts from fMRI data. *Neuroimage*. 2015;112:267-277. doi:10.1016/j.neuroimage.2015.02.064

15. Bijsterbosch J, Harrison S, Duff E, Alfaro-Almagro F, Woolrich M, Smith S. Investigations into within- and between-subject resting-state amplitude variations. *NeuroImage*. 2017;159:57-69. doi:10.1016/j.neuroimage.2017.07.014

16. Alfaro-Almagro F, Jenkinson M, Bangerter NK, et al. Image processing and Quality Control for the first 10,000 brain imaging datasets from UK Biobank. *NeuroImage*. 2018;166:400-424. doi:10.1016/j.neuroimage.2017.10.034

17. Smith SM, Fox PT, Miller KL, et al. Correspondence of the brain’s functional architecture during activation and rest. *Proc Natl Acad Sci*. 2009;106(31):13040-13045. doi:10.1073/pnas.0905267106

18. Yeo BTT, Krienen FM, Sepulcre J, et al. The organization of the human cerebral cortex estimated by intrinsic functional connectivity. *Journal of Neurophysiology*. 2011;106(3):1125-1165. doi:10.1152/jn.00338.2011

19. Nickerson LD, Smith SM, Öngür D, Beckmann CF. Using Dual Regression to Investigate Network Shape and Amplitude in Functional Connectivity Analyses. *Front Neurosci*. 2017;11:115. doi:10.3389/fnins.2017.00115

20. Meng X, Jiang R, Lin D, et al. Predicting individualized clinical measures by a generalized prediction framework and multimodal fusion of MRI data. *NeuroImage*. 2017;145(Part B):218-229. doi:10.1016/j.neuroimage.2016.05.026

21. Li H, Smith SM, Gruber S, et al. Denoising scanner effects from multimodal MRI data using linked independent component analysis. *NeuroImage*. Published online November 22, 2019:116388. doi:10.1016/j.neuroimage.2019.116388

22. Epskamp S, Waldorp LJ, Mõttus R, Borsboom D. The Gaussian Graphical Model in Cross-Sectional and Time-Series Data. *Multivariate Behav Res*. 2018;53(4):453-480. doi:10.1080/00273171.2018.1454823

23. Koller D, Friedman N. *Probabilistic Graphical Models: Principles and Techniques*. MIT Press; 2009.

24. Blondel VD, Guillaume JL, Lambiotte R, Lefebvre E. Fast unfolding of communities in large networks. *J Stat Mech*. 2008;2008(10):P10008. doi:2008101003130400

25. Newman MEJ, Girvan M. Finding and evaluating community structure in networks. *Phys Rev E*. 2004;69(2):026113. doi:10.1103/PhysRevE.69.026113

26. Newman MEJ. *Networks: An Introduction*. Oxford University Press; 2010.

27. Guimerà R, Nunes Amaral LA. Functional cartography of complex metabolic networks. *Nature*. 2005;433(7028):895-900. doi:10.1038/nature03288

28. Burnham KP, Anderson DR, eds. *Model Selection and Multimodel Inference*. Springer; 2004. doi:10.1007/b97636

29. Lüdecke D, Ben-Shachar MS, Patil I, Waggoner P, Makowski D. performance: An R Package for Assessment, Comparison and Testing of Statistical Models. *Journal of Open Source Software*. 2021;6(60):3139. doi:10.21105/joss.03139
